# Digital CRISPR/Cas‐Assisted Assay for Rapid and Sensitive Detection of SARS‐CoV‐2

**DOI:** 10.1002/advs.202003564

**Published:** 2021-01-12

**Authors:** Joon Soo Park, Kuangwen Hsieh, Liben Chen, Aniruddha Kaushik, Alexander Y. Trick, Tza‐Huei Wang

**Affiliations:** ^1^ Department of Biomedical Engineering Johns Hopkins University Baltimore MD 21218 USA; ^2^ Department of Mechanical Engineering Johns Hopkins University Baltimore MD 21218 USA; ^3^ Institute for NanoBioTechnology Johns Hopkins University Baltimore MD 21218 USA

**Keywords:** CRISPR/Cas, diagnostics, digital, nucleic acid, SARS‐CoV‐2

## Abstract

The unprecedented demand for rapid diagnostics in response to the COVID‐19 pandemic has brought the spotlight onto clustered regularly interspaced short palindromic repeats (CRISPR)/CRISPR‐associated systems (Cas)‐assisted nucleic acid detection assays. Already benefitting from an elegant detection mechanism, fast assay time, and low reaction temperature, these assays can be further advanced via integration with powerful, digital‐based detection. Thus motivated, the first digital CRISPR/Cas‐assisted assay—coined digitization‐enhanced CRISPR/Cas‐assisted one‐pot virus detection (deCOViD)—is developed and applied toward SARS‐CoV‐2 detection. deCOViD is realized through tuning and discretizing a one‐step, fluorescence‐based, CRISPR/Cas12a‐assisted reverse transcription recombinase polymerase amplification assay into sub‐nanoliter reaction wells within commercially available microfluidic digital chips. The uniformly elevated digital concentrations enable deCOViD to achieve qualitative detection in <15 min and quantitative detection in 30 min with high signal‐to‐background ratio, broad dynamic range, and high sensitivity—down to 1 genome equivalent (GE) µL^−1^ of SARS‐CoV‐2 RNA and 20 GE µL^−1^ of heat‐inactivated SARS‐CoV‐2, which outstrips its benchtop‐based counterpart and represents one of the fastest and most sensitive CRISPR/Cas‐assisted SARS‐CoV‐2 detection to date. Moreover, deCOViD can detect RNA extracts from clinical samples. Taken together, deCOViD opens a new avenue for advancing CRISPR/Cas‐assisted assays and combating the COVID‐19 pandemic and beyond.

## Graphical abstract

A digital clustered regularly interspaced short palindromic repeats (CRISPR)/Cas‐assisted nucleic acid detection assay is created by discretizing a one‐step, fluorescence‐based, CRISPR/Cas12a‐assisted reverse transcription recombinase polymerase amplification assay within sub‐nanoliter reaction wells of a commercial microfluidic digital chip, which enables quantitative detection of 1 genome equivalent µL^−1^ of SARS‐CoV‐2 RNA in <30 min—one of the fastest and most sensitive CRISPR/Cas‐assisted detection to date.

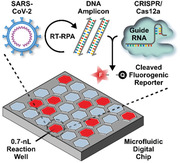

The crucial but unmet need for rapid and sensitive nucleic acid detection assays for diagnostic testing of highly infectious diseases has become front and center as the COVID‐19 pandemic^[^ ^1^, ^2^, ^3^ ^]^ continues to devastate. The global scientific community has responded with unprecedented urgency to develop assays that can rapidly and sensitively detect the causative SARS‐CoV‐2 virus and curb the spread of COVID‐19.^[^ ^4^, ^5^, ^6^ ^]^ As a result, a plethora of assays,^[^ ^7^, ^8^, ^9^, ^10^, ^11^ ^]^ including standardized assays based on reverse transcription PCR (RT‐PCR)^[^ ^12^, ^13^ ^]^ and emerging isothermal assays based on reverse transcription loop mediated isothermal amplification (RT‐LAMP),^[^ ^14^, ^15^, ^16^, ^17^, ^18^, ^19^, ^20^, ^21^ ^]^ have been reported within a remarkably short period of time. Among the emerging assays, those incorporating clustered regularly interspaced short palindromic repeats (CRISPR)/CRISPR‐associated systems (Cas)^[^ ^22^, ^23^, ^24^, ^25^, ^26^, ^27^, ^28^, ^29^, ^30^, ^31^, ^32^ ^]^ (e.g., DETECTR^[^ ^22^ ^]^ and SHERLOCK^[^ ^31^, ^32^ ^]^) have attracted particular attention due to their elegant detection mechanism, fast turnaround time, and potential circumvention of instrument‐intensive thermocycling. However, despite these advantages and rapid advances, with the exception of two recently reported one‐step CRISPR/Cas‐assisted assays,^[^ ^23^, ^31^ ^]^ current CRISPR/Cas‐assisted assays remain hampered by a requisite but separate preamplification step. The resulting multipot and multistep assay format has precluded them from digital detection—a powerful detection approach that has enhanced the diagnostic capabilities of PCR^[^ ^33^, ^34^, ^35^, ^36^ ^]^ and LAMP.^[^ ^37^ ^]^ Indeed, to date, no CRISPR/Cas‐assisted assay has been implemented in digital detection format, nor have the potential enhancements from digitization been realized or explored.

In response, we have developed digitization‐enhanced CRISPR/Cas‐assisted one‐pot virus detection (deCOViD)—the first digital CRISPR/Cas‐assisted assay that can detect SARS‐CoV‐2 RNA and heat‐inactivated SARS‐CoV‐2 (**Figure** **1**). In deCOViD, SARS‐CoV‐2 RNA and inactivated SARS‐CoV‐2 can be fluorescently detected by a single‐step assay that integrates reverse transcription, recombinase polymerase amplification (RPA),^[^ ^38^ ^]^ and CRSIPR/Cas12a‐based detection.^[^ ^39^ ^]^ Within the assay, RNA targets are reverse transcribed and amplified via RT‐RPA into DNA amplicons, which activate Cas12a‐guide RNA complexes to cleave single‐stranded DNA fluorogenic reporters and yield fluorescence.^[^ ^39^ ^]^ This single‐step assay is then fine‐tuned to ensure that it can be easily loaded into commercially available microfluidic digital chips and reliably discretized within digital reaction wells. Upon assay digitization within digital reaction wells, every copy of the target is isolated at a locally elevated concentration, facilitating rapid amplification that is independent of the sample concentration. This advantageous mechanism therefore empowers deCOViD to outperform its bulk counterpart in detecting SARS‐CoV‐2 and potentially other RNA and even DNA targets with accelerated detection time, enhanced signal‐to‐background ratio, widened dynamic range, and improved sensitivity.

**Figure 1:**
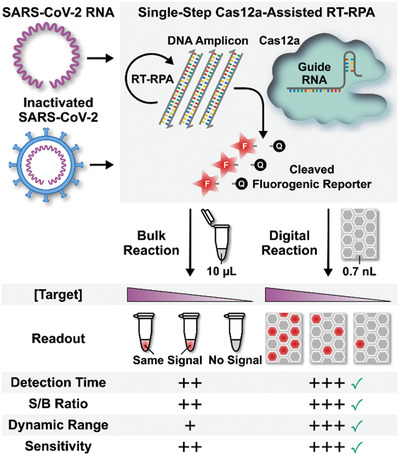
Overview of digitization‐enhanced CRISPR/Cas‐assisted one‐pot virus detection (deCOViD). SARS‐CoV‐2 RNA and inactivated SARS‐CoV‐2 virus can be detected by a single‐step CRISPR/Cas12a‐assisted reverse transcription recombinase polymerase amplification (RT‐RPA) assay, which produces DNA amplicons from RNA targets to activate Cas12a‐based cleavage of fluorogenic reporters in a single step. Importantly, compared to microliter‐scale bulk assay, deCOViD takes advantage of assay digitization to elevate the RNA concentration in sub‐nanoliter digital reaction wells and facilitate rapid amplification, leading to faster detection time for low target concentrations, higher signal‐to‐background (S/B) ratio, wider dynamic range, and better sensitivity.

We first established a prerequisite bulk‐based single‐step CRISPR/Cas12a‐assisted RT‐RPA assay that can detect SARS‐CoV‐2. We adopted the RPA primers and the Cas12a guide RNAs (Table S1, Supporting Information) from a recently reported one‐pot CRISPR/Cas12a‐assisted RT‐RPA assay that uses two Cas12a guide RNAs to detect a fragment of the SARS‐CoV‐2 N gene^[^ ^23^ ^]^ and employed Alexa647‐labeled fluorogenic reporter from our previous work.^[^ ^40^ ^]^ We elected to test this assay against standardized synthetic SARS‐CoV‐2 RNA from NIAID BEI Resources (NR‐52358) rather than an RNA sequence in vitro transcribed in house. Performed in a benchtop real‐time qPCR instrument under an isothermal condition, this bulk assay successfully detected 200, 100, and 50 genome equivalents (GE) µL^−1^ RNA, as indicated by strong fluorescence signals above the no‐RNA control (i.e., 0 GE µL^−1^) at 60 min (**Figure** **2**a). In addition to detecting the standardized SARS‐CoV‐2 RNA for the first time, we made two notable improvements for this bulk assay. First, we elevated the reaction temperature to 42 °C (Figure S1a, Supporting Information) to improve reverse transcription efficiency in the assay. We also added 0.01 mg mL^−1^ bovine serum albumin to our assay (Figure S1b, Supporting Information) to reduce adsorption of enzymes to reaction tube walls.

**Figure 2:**
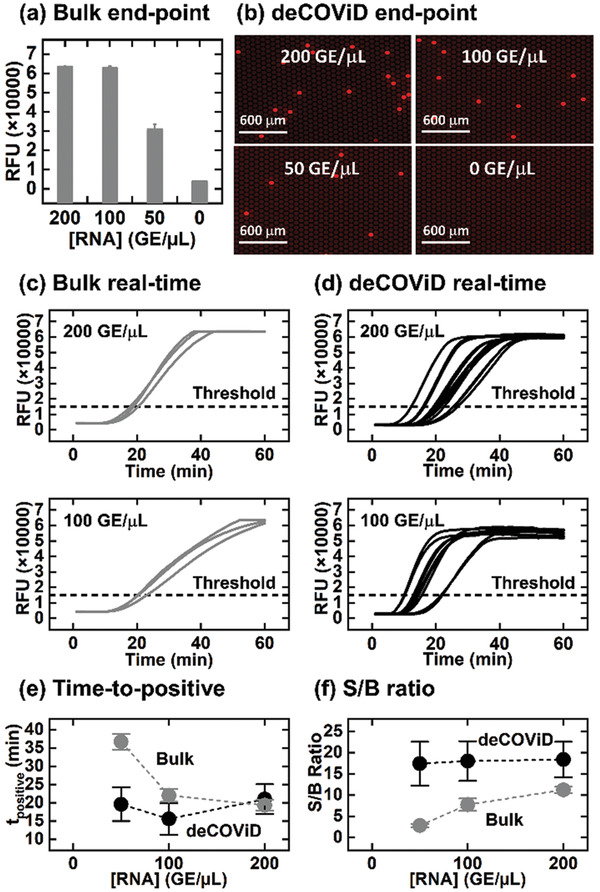
Comparison of bulk CRISPR/Cas12a‐assisted RT‐RPA and deCOViD. Detection of various concentrations of SARS‐CoV‐2 RNA (in genome equivalent (GE) µL^−1^) is successful at 60 min via both a) bulk CRISPR/Cas12a‐assisted RT‐RPA, which yields higher fluorescence signals than the no‐RNA control, and b) deCOViD (i.e., digital CRISPR/Cas12a‐assisted RT‐RPA), which outputs serially reduced number of positive reaction wells with strong fluorescence and no positive reaction wells for the no‐RNA control. Real‐time amplifications curves from samples with 200 and 100 GE µL^−1^ RNA reveal that c) the bulk assay speed decreases from the reduced sample concentration, but d) deCOViD maintains consistent assay speed from both sample RNA concentrations, while also allowing qualitative detection in <15 min by detecting the early positive reaction wells. Compared to the bulk assay, assay digitization in deCOViD facilitates e) faster and RNA concentration‐independent time‐to‐positive (i.e., the time at which the sample fluorescence signal surpasses the predefined threshold) and f) higher signal‐to‐background (S/B) ratios at 30 min reaction time across various sample concentrations. Data in (a), (e), and (f) presented as mean ± SD, *n* = 3 (bulk) or *n* > 3 (deCOViD).

For actualizing deCOViD, we digitized the bulk CRISPR/Cas12a‐assisted RT‐RPA assay in QuantStudio 3D Digital PCR 20K Chip with 0.7 nL digital reaction wells. QuantStudio digital chip offers an attractive option for assay digitization thanks to its commercial availability, simple and fast digitization workflow, and compatibility with fluorescence microscopy—our preferred detection method for this proof‐of‐concept study. Robust assay loading and discretization into QuantStudio chips could be completed with ≈5 min hands‐on time per chip, and was further facilitated by adding 0.1% Tween‐20 in the reaction mix, which reduced its viscosity without compromising its detection performance (Figure S2, Supporting Information). We also verified that no amplification would occur at room temperature during assay digitization (Figure S3, Supporting Information). After digitizing 200, 100, and 50 GE µL^−1^ RNA into QuantStudio chips and heating at 42 °C for 60 min, we detected digital reaction wells with strong fluorescence (i.e., positive) via fluorescence microscopy. Importantly, we found fewer positive reaction wells as the RNA concentration decreased, indicating that single copies of RNA were indeed digitized and amplified (Figure 2b and Figure S4, Supporting Information). Finally, we detected 0 positive reaction well from the no‐RNA control. These results offer strong initial validation for deCOViD.

We subsequently acquired and compared real‐time amplification curves from both the bulk assay and deCOViD at different RNA concentrations to illustrate the benefit of assay digitization. For the bulk assay, we used the benchtop real‐time qPCR instrument to acquire the amplification curves. Samples with 200 RNA GE µL^−1^ yielded amplification curves that plateaued at ≈40 min, whereas samples with 100 RNA GE µL^−1^ yielded noticeably flatter amplification curves that plateaued at ≈60 min (Figure 2c). These results suggest that the bulk reaction rate slows with reduced RNA concentration. For deCOViD, we employed a custom‐assembled miniature heater that is compatible with fluorescence microscopy (Figure S5, Supporting Information) to simultaneously measure the fluorescence intensities from ≈800 digital reaction wells as a function of time (Figure S6, Supporting Information). All resulting deCOViD amplification curves from both 200 and 100 RNA GE µL^−1^ rose sharply and plateaued in ≈40 min, suggesting comparable amplification speeds between the two target concentrations (Figure 2d). The contrasting bulk and deCOViD results illustrate that, by digitizing each RNA molecule within a digital reaction well at a uniform concentration, the amplification speed of deCOViD becomes independent of the sample RNA concentration.

Quantitative analysis of real‐time detection results further confirms the advantages of deCOViD in amplification speed and signal‐to‐background ratio. As the RNA concentration decreased from 200, 100 to 50 GE µL^−1^, the average time‐to‐positive (i.e., the time at which the sample fluorescence signal surpasses the predefined threshold) for the bulk assay increased from 19.3 ± 1.5, 22.0 ± 1.7 to 36.7 ± 2.1 min (Figure 2e and Figure S7a, Supporting Information). In contrast, the average time‐to‐positive for deCOViD remained consistent across the three RNA concentrations at 21.0 ± 4.1, 15.6 ± 4.3, and 19.6 ± 4.6 min (Figure 2e and Figure S7b, Supporting Information). These results show that deCOViD retains consistently rapid detection even as the RNA concentration decreases. This advantage is especially useful for qualitative detection, which could be achieved in <15 min—as soon as the first few reaction wells became positive—even for 50 RNA GE µL^−1^ (Figure S7b, Supporting Information). We further note that, as positive reaction wells across different RNA concentrations were all detectable within 30 min (Figure S8, Supporting Information), we could shorten the assay time to 30 min for quantitative detection and deCOViD could still retain higher signal‐to‐background ratio than the bulk assay and achieve robust detection (Figure 2f).

With the shortened 30 min assay time, we proceeded to demonstrate improved detection capability of deCOViD by challenging both the bulk assay and deCOViD with titrations of synthetic SARS‐CoV‐2 RNA. Relying on fluorescence intensity measurements, the bulk assay could detect down to 10 GE µL^−1^ (**Figure** **3**a, Bulk). On the other hand, deCOViD quantifies the fraction of positive reaction wells (i.e., percent positive) and was able to detect 1 GE µL^−1^ (equivalent to ≈15 GE), or a tenfold improvement in the limit of detection (Figure 3a, deCOViD). The speed and sensitivity of deCOViD outstrip most CRISPR/Cas‐assisted SARS‐CoV‐2 assays, including DETECTR^[^ ^22^ ^]^ and SHERLOCK^[^ ^31^, ^32^ ^]^ (Table S2, Supporting Information). Of note, we found that deCOViD was ≈4–5‐fold less sensitive than digital RT‐PCR (Figure S9, Supporting Information), but its 30 min assay time and isothermal reaction condition significantly offer significant advantages. Moreover, deCOViD widened the dynamic range for quantitative detection. The signal from the bulk assay saturated at ≈280 GE µL^−1^ (Figure S10a, Supporting Information), rendering quantitative detection above this concentration impossible. On the other hand, deCOViD displayed linearly correlated detection beyond 500 GE µL^−1^ (Figure S10b, Supporting Information). These results show that deCOViD enhances the assay sensitivity, quantification, and dynamic range—a clear illustration for the benefits of digitization.

**Figure 3:**
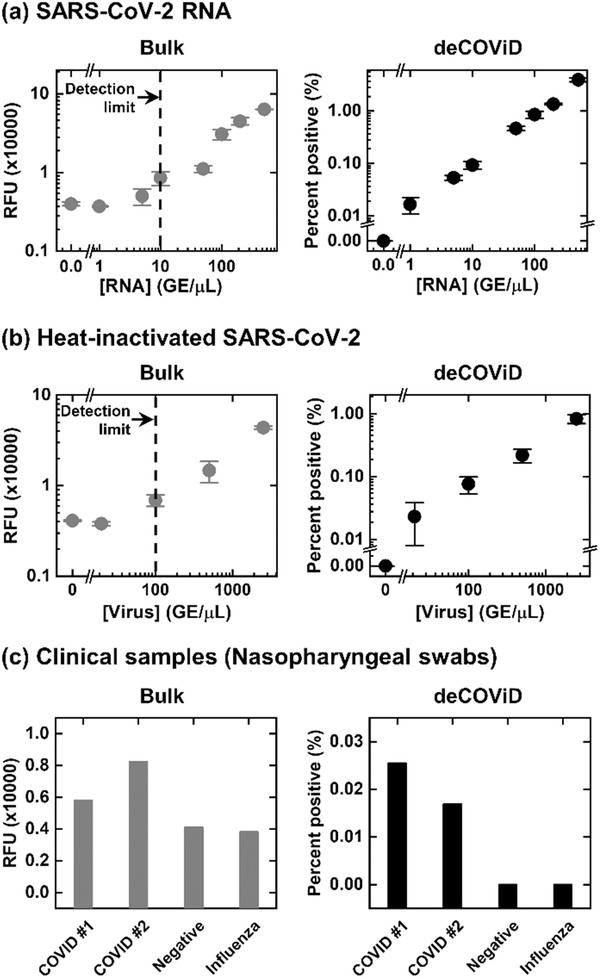
Detection of SARS‐CoV‐2 RNA, heat‐inactivated SARS‐CoV‐2, and clinical samples in 30 min. a) In detecting SARS‐CoV‐2 RNA, the bulk assay can detect ten genome equivalent (GE) µL^−1^, while deCOViD can detect 1 GE µL^−1^, or a tenfold improvement. This RNA detection sensitivity also outstrips existing CRISPR/Cas‐assisted assays. b) In detecting heat‐inactivated SARS‐CoV‐2, the bulk assay can detect 100 GE µL^−1^, while deCOViD can detect 20 GE µL^−1^, or a fivefold improvement. Notably, both assays can readily detect heat‐inactivated SARS‐CoV‐2 without RNA extraction. c) When testing RNA extracts from clinical nasopharyngeal swabs, both the bulk assay and deCOViD can differentiate positive samples from the negative sample and the influenza sample. Data in (a) and (b) presented as mean ± SD, *n* = 3.

We also showed that deCOViD outperformed the bulk assay in directly detecting heat‐inactivated SARS‐CoV‐2 without RNA extraction. For this demonstration, we spiked 2500, 500, 100, and 20 GE µL^−1^ of heat‐inactivated SARS‐CoV‐2 from NIAID BEI Resources (NR‐52350) directly into both the bulk assay and deCOViD. The bulk assay detected 100 GE µL^−1^ (Figure 3b, bulk), while deCOViD detected 20 GE µL^−1^ (Figure 3b, deCOViD), or a fivefold improvement. We note that heat‐inactivated SARS‐CoV‐2 was directly detected by both the bulk assay and deCOViD without any preparatory RNA extraction steps. Our results reveal the feasibility of directly detecting heat‐inactivated SARS‐CoV‐2 with a CRISPR/Cas‐assisted assay, a capability similar to previously reported RNA extraction‐free detection of heat‐inactivated SARS‐CoV‐2 via RT‐PCR.^[^ ^41^ ^]^

Finally, we demonstrated the feasibility of clinical sample testing. For this initial demonstration, we tested four RNA extracts from clinical nasopharyngeal swabs via both the bulk assay and deCOViD. The four samples, including two SARS‐CoV‐2 positive samples, one SARS‐CoV‐2 negative sample, and one influenza sample, were first confirmed via an in‐house RT‐qPCR assay that uses the US CDC‐approved SARS‐CoV‐2 primers and probes^[^ ^42^ ^]^ (Figure S11, Supporting Information). Both the bulk assay and deCOViD yielded higher signals from the two positive samples than those from the negative sample—indicating successful detection results that agree with RT‐qPCR (Figure 3c). In addition, for the influenza sample, both the bulk assay and deCOViD yielded signals that are indistinguishable from those from the negative sample, which matches RT‐qPCR while also illustrating the specificity of both methods (Figure 3c and Figure S11, Supporting Information). Finally, as a demonstration, we diluted one of the positive samples by twofold and challenged both the bulk assay and deCOViD. This diluted sample became undetectable to the bulk assay but was detected by deCOViD (Figure S12, Supporting Information). These results thus provide additional support for the improved sensitivity of deCOViD over the bulk assay.

In conclusion, we have developed the first digital CRISPR/Cas‐assisted assay, deCOViD, that can rapidly and sensitively detect both SARS‐CoV‐2 RNA and heat‐inactivated SARS‐CoV‐2. Successful digitization of a fine‐tuned single‐step CRISPR/Cas12a‐assisted RT‐RPA assay in commercial QuantStudio digital chip, coupled to successful development of digital real‐time detection capability by incorporating a miniature heater, allows us to realize deCOViD and demonstrate enhancements in assay time, signal‐to‐background ratio, dynamic range, and sensitivity over the bulk assay. As a result, deCOViD can accelerate qualitative detection to <15 min and achieve quantitative detection down to 1 GE µL^−1^ RNA and 20 GE µL^−1^ heat‐inactivated SARS‐CoV‐2 without RNA extraction in 30 min—among the fastest and the most sensitive CRISPR/Cas‐assisted SARS‐CoV‐2 assays to date. In addition to building upon our initial demonstration of clinical sample testing and conducting a more thorough clinical validation, we see several routes for advancing deCOViD. First, we can further improve the speed and sensitivity of deCOViD by optimizing the concentrations of RPA primers, Cas12a effector, Cas12‐guide RNAs, and fluorogenic reporter in custom digital chips with greater total analysis volume and rapid digitization workflow.^[^ ^43^, ^44^, ^45^ ^]^ We also envision enhancing the user‐friendliness of deCOViD by predigitizing and drying magnesium acetate (the chemical that initiates RPA) in digital chips.^[^ ^46^ ^]^ Second, we can replace fluorescence microscopy with mobile phone‐based fluorescence detection^[^ ^47^, ^48^ ^]^ and couple to our miniature heater to make a portable device with the potential for point‐of‐care use. Third, as the relationship between SARS‐CoV‐2 viral load and COVID‐19 state remains incompletely understood, we suspect that our highly quantitative deCOViD may provide a new tool in such studies. Finally, as deCOViD can be readily designed for other DNA or RNA targets, we foresee applying deCOViD toward other diseases. Based on the encouraging results and potential for improvement, we believe deCOViD can open a new avenue for advancing CRISPR/Cas‐assisted diagnostic assays and provide a new tool for combating the COVID‐19 pandemic and beyond.

## Conflict of Interest

The authors declare no conflict of interest.

## Supporting information

Supporting Information

## References

[1] A. S. Fauci , H. C. Lane , R. R. Redfield , N. Engl. J. Med. 2020, 382, 1268. 32109011 10.1056/NEJMe2002387 PMC7121221

[2] C. Wang , P. W. Horby , F. G. Hayden , G. F. Gao , Lancet 2020, 395, 470. 31986257 10.1016/S0140-6736(20)30185-9 PMC7135038

[3] N. Zhu , D. Zhang , W. Wang , X. Li , B. Yang , J. Song , X. Zhao , B. Huang , W. Shi , R. Lu , P. Niu , F. Zhan , X. Ma , D. Wang , W. Xu , G. Wu , G. F. Gao , W. Tan , N. Engl. J. Med. 2020, 382, 727. 31978945 10.1056/NEJMoa2001017 PMC7092803

[4] J. Bedford , D. Enria , J. Giesecke , D. L. Heymann , C. Ihekweazu , G. Kobinger , H. C. Lane , Z. Memish , M.‐D. Oh , A. A. Sall , A. Schuchat , K. Ungchusak , L. H. Wieler , Lancet 2020, 395, 1015. 32197103 10.1016/S0140-6736(20)30673-5 PMC7270596

[5] M. N. Esbin , O. N. Whitney , S. Chong , A. Maurer , X. Darzacq , R. Tjian , RNA 2020, 26, 771. 32358057 10.1261/rna.076232.120 PMC7297120

[6] R. Weissleder , H. Lee , J. Ko , M. J. Pittet , Sci. Transl. Med. 2020, 12, eabc1931. 32493791 10.1126/scitranslmed.abc1931

[7] L. J. Carter , L. V. Garner , J. W. Smoot , Y. Li , Q. Zhou , C. J. Saveson , J. M. Sasso , A. C. Gregg , D. J. Soares , T. R. Beskid , S. R. Jervey , C. Liu , ACS Cent. Sci. 2020, 6, 591. 32382657 10.1021/acscentsci.0c00501 PMC7197457

[8] W. Feng , A. M. Newbigging , C. Le , B. Pang , H. Peng , Y. Cao , J. Wu , G. Abbas , J. Song , D.‐B. Wang , M. Cui , J. Tao , D. L. Tyrrell , X.‐E. Zhang , H. Zhang , X. C. Le , Anal. Chem. 2020, 92, 10196. 32573207 10.1021/acs.analchem.0c02060

[9] T. Ji , Z. Liu , G. Wang , X. Guo , S. A. khan , C. Lai , H. Chen , S. Huang , S. Xia , B. Chen , H. Jia , Y. Chen , Q. Zhou , Biosens. Bioelectron. 2020, 166, 112455. 32739797 10.1016/j.bios.2020.112455 PMC7371595

[10] Y.‐W. Tang , J. E. Schmitz , D. H. Persing , C. W. Stratton , J. Clin. Microbiol. 2020, 58, e00512. 32245835 10.1128/JCM.00512-20 PMC7269383

[11] B. Udugama , P. Kadhiresan , H. N. Kozlowski , A. Malekjahani , M. Osborne , V. Y. C. Li , H. Chen , S. Mubareka , J. B. Gubbay , W. C. W. Chan , ACS Nano 2020, 14, 3822. 32223179 10.1021/acsnano.0c02624

[12] D. K. W. Chu , Y. Pan , S. M. S. Cheng , K. P. Y. Hui , P. Krishnan , Y. Liu , D. Y. M. Ng , C. K. C. Wan , P. Yang , Q. Wang , M. Peiris , L. L. M. Poon , Clin. Chem. 2020, 66, 549. 32031583 10.1093/clinchem/hvaa029 PMC7108203

[13] V. M. Corman , O. Landt , M. Kaiser , R. Molenkamp , A. Meijer , D. K. Chu , T. Bleicker , S. Brünink , J. Schneider , M. L. Schmidt , D. G. Mulders , B. L. Haagmans , B. van der Veer , S. van den Brink , L. Wijsman , G. Goderski , J.‐L. Romette , J. Ellis , M. Zambon , M. Peiris , H. Goossens , C. Reusken , M. P. Koopmans , C. Drosten , Eurosurveillance 2020, 25, 2000045.

[14] Y. H. Baek , J. Um , K. J. C. Antigua , J.‐H. Park , Y. Kim , S. Oh , Y.‐I. Kim , W.‐S. Choi , S. G. Kim , J. H. Jeong , B. S. Chin , H. D. G. Nicolas , J.‐Y. Ahn , K. S. Shin , Y. K. Choi , J.‐S. Park , M.‐S. Song , Emerging Microbes Infect. 2020, 9, 998. 10.1080/22221751.2020.1756698 PMC7301696 32306853

[15] V. L. D. Thi , K. Herbst , K. Boerner , M. Meurer , L. P. M. Kremer , D. Kirrmaier , A. Freistaedter , D. Papagiannidis , C. Galmozzi , M. L. Stanifer , S. Boulant , S. Klein , P. Chlanda , D. Khalid , I. B. Miranda , P. Schnitzler , H.‐G. Kräusslich , M. Knop , S. Anders , Sci. Transl. Med. 2020, 12, eabc7075. 32719001 10.1126/scitranslmed.abc7075 PMC7574920

[16] W. E. Huang , B. Lim , C.‐C. Hsu , D. Xiong , W. Wu , Y. Yu , H. Jia , Y. Wang , Y. Zeng , M. Ji , H. Chang , X. Zhang , H. Wang , Z. Cui , Microb. Biotechnol. 2020, 13, 950. 32333644 10.1111/1751-7915.13586 PMC7264870

[17] J. Y. H. Lee , N. Best , J. McAuley , J. L. Porter , T. Seemann , M. B. Schultz , M. Sait , N. Orlando , K. Mercoulia , S. A. Ballard , J. Druce , T. Tran , M. G. Catton , M. J. Pryor , H. L. Cui , A. Luttick , S. McDonald , A. Greenhalgh , J. C. Kwong , N. L. Sherry , M. Graham , T. Hoang , M. Herisse , S. J. Pidot , D. A. Williamson , B. P. Howden , I. R. Monk , T. P. Stinear , bioRxiv 2020, 2020.04.28.067363.

[18] G.‐S. Park , K. Ku , S.‐H. Baek , S.‐J. Kim , S. I. Kim , B.‐T. Kim , J.‐S. Maeng , J. Mol. Diagn. 2020, 22, 729. 32276051 10.1016/j.jmoldx.2020.03.006 PMC7144851

[19] C. Yan , J. Cui , L. Huang , B. Du , L. Chen , G. Xue , S. Li , W. Zhang , L. Zhao , Y. Sun , H. Yao , N. Li , H. Zhao , Y. Feng , S. Liu , Q. Zhang , D. Liu , J. Yuan , Clin. Microbiol. Infect. 2020, 26, 773. 32276116 10.1016/j.cmi.2020.04.001 PMC7144850

[20] L. Yu , S. Wu , X. Hao , X. Dong , L. Mao , V. Pelechano , W.‐H. Chen , X. Yin , Clin. Chem. 2020, 66, 975. 32315390 10.1093/clinchem/hvaa102 PMC7188121

[21] A. Ganguli , A. Mostafa , J. Berger , M. Y. Aydin , F. Sun , S. A. S. d. Ramirez , E. Valera , B. T. Cunningham , W. P. King , R. Bashir , Proc. Natl. Acad. Sci. USA 2020, 202014739. 10.1073/pnas.2014739117 PMC7502724 32868442

[22] J. P. Broughton , X. Deng , G. Yu , C. L. Fasching , V. Servellita , J. Singh , X. Miao , J. A. Streithorst , A. Granados , A. Sotomayor‐Gonzalez , K. Zorn , A. Gopez , E. Hsu , W. Gu , S. Miller , C.‐Y. Pan , H. Guevara , D. A. Wadford , J. S. Chen , C. Y. Chiu , Nat. Biotechnol. 2020, 38, 870. 32300245 10.1038/s41587-020-0513-4 PMC9107629

[23] X. Ding , K. Yin , Z. Li , R. V. Lalla , E. Ballesteros , M. M. Sfeir , C. Liu , Nat. Commun. 2020, 11, 4711. 32948757 10.1038/s41467-020-18575-6 PMC7501862

[24] L. Guo , X. Sun , X. Wang , C. Liang , H. Jiang , Q. Gao , M. Dai , B. Qu , S. Fang , Y. Mao , Y. Chen , G. Feng , Q. Gu , R. R. Wang , Q. Zhou , W. Li , Cell Discovery 2020, 6, 34. 32435508 10.1038/s41421-020-0174-y PMC7235268

[25] Z. Huang , D. Tian , Y. Liu , Z. Lin , C. J. Lyon , W. Lai , D. Fusco , A. Drouin , X. Yin , T. Hu , B. Ning , Biosens. Bioelectron. 2020, 164, 112316. 32553350 10.1016/j.bios.2020.112316 PMC7245202

[26] C. Lucia , P.‐B. Federico , G. C. Alejandra , bioRxiv 2020, 2020.02.29.971127.

[27] K. H. Ooi , J. W. D. Tay , S. Y. Teo , M. M. Liu , P. Kaewsapsak , S. Jin , Y.‐G. Gao , M. H. Tan , bioRxiv 2020, 2020.07.03.185850.

[28] X. Wang , M. Zhong , Y. Liu , P. Ma , L. Dang , Q. Meng , W. Wan , X. Ma , J. Liu , G. Yang , Z. Yang , X. Huang , M. Liu , Sci. Bull. 2020, 65, 1436. 10.1016/j.scib.2020.04.041 PMC7198415 32373393

[29] K. Yoshimi , K. Takeshita , S. Yamayoshi , S. Shibumura , Y. Yamauchi , M. Yamamoto , H. Yotsuyanagi , Y. Kawaoka , T. Mashimo , medRxiv 2020, 2020.06.02.20119875. 10.1016/j.isci.2022.103830 PMC8801231 35128347

[30] T. Hou , W. Zeng , M. Yang , W. Chen , L. Ren , J. Ai , J. Wu , Y. Liao , X. Gou , Y. Li , X. Wang , H. Su , B. Gu , J. Wang , T. Xu , PLoS Pathog. 2020, 16, e1008705. 32853291 10.1371/journal.ppat.1008705 PMC7451577

[31] J. Joung , A. Ladha , M. Saito , N.‐G. Kim , A. E. Woolley , M. Segel , R. P. J. Barretto , A. Ranu , R. K. Macrae , G. Faure , E. I. Ioannidi , R. N. Krajeski , R. Bruneau , M.‐L. W. Huang , X. G. Yu , J. Z. Li , B. D. Walker , D. T. Hung , A. L. Greninger , K. R. Jerome , J. S. Gootenberg , O. O. Abudayyeh , F. Zhang , N. Engl. J. Med. 2020, 383, 1492. 32937062 10.1056/NEJMc2026172 PMC7510942

[32] M. Patchsung , K. Jantarug , A. Pattama , K. Aphicho , S. Suraritdechachai , P. Meesawat , K. Sappakhaw , N. Leelahakorn , T. Ruenkam , T. Wongsatit , N. Athipanyasilp , B. Eiamthong , B. Lakkanasirorat , T. Phoodokmai , N. Niljianskul , D. Pakotiprapha , S. Chanarat , A. Homchan , R. Tinikul , P. Kamutira , K. Phiwkaow , S. Soithongcharoen , C. Kantiwiriyawanitch , V. Pongsupasa , D. Trisrivirat , J. Jaroensuk , T. Wongnate , S. Maenpuen , P. Chaiyen , S. Kamnerdnakta , J. Swangsri , S. Chuthapisith , Y. Sirivatanauksorn , C. Chaimayo , R. Sutthent , W. Kantakamalakul , J. Joung , A. Ladha , X. Jin , J. S. Gootenberg , O. O. Abudayyeh , F. Zhang , N. Horthongkham , C. Uttamapinant , Nat. Biomed. Eng. 2020. 10.1038/s41551-020-00603-x 32848209

[33] R. H. Sedlak , K. R. Jerome , Expert Rev. Mol. Diagn. 2014, 14, 501. 24724628 10.1586/14737159.2014.910456

[34] J. Kuypers , K. R. Jerome , J. Clin. Microbiol. 2017, 55, 1621. 28298452 10.1128/JCM.00211-17 PMC5442518

[35] P. L. Quan , M. Sauzade , E. Brouzes , Sensors 2018, 18, 1271. 10.3390/s18041271 PMC5948698 29677144

[36] S. J. Salipante , K. R. Jerome , Clin. Chem. 2020, 66, 117. 31704712 10.1373/clinchem.2019.304048

[37] H. Yuan , Y. Chao , H. C. Shum , Small 2020, 16, 1904469. 10.1002/smll.201904469 31899592

[38] O. Piepenburg , C. H. Williams , D. L. Stemple , N. A. Armes , PLoS Biol. 2006, 4, e204. 16756388 10.1371/journal.pbio.0040204 PMC1475771

[39] J. S. Chen , E. B. Ma , L. B. Harrington , M. Da Costa , X. R. Tian , J. M. Palefsky , J. A. Doudna , Science 2018, 360, 436. 29449511 10.1126/science.aar6245 PMC6628903

[40] K. Hsieh , G. Zhao , T.‐H. Wang , Analyst 2020, 145, 4880. 32478351 10.1039/d0an00664e PMC7362986

[41] N. Lubke , T. Senff , S. Scherger , S. Hauka , M. Andree , O. Adams , J. Timm , A. Walker , J. Clin. Virol. 2020, 130, 104579. 32795959 10.1016/j.jcv.2020.104579 PMC7405857

[42] X. Lu , L. Wang , S. K. Sakthivel , B. Whitaker , J. Murray , S. Kamili , B. Lynch , L. Malapati , S. A. Burke , J. Harcourt , A. Tamin , N. J. Thornburg , J. M. Villanueva , S. Lindstrom , Emerging Infect. Dis. 2020, 26, 1654. 10.3201/eid2608.201246 PMC7392423 32396505

[43] K. Hsieh , H. C. Zec , L. Chen , A. M. Kaushik , K. E. Mach , J. C. Liao , T.‐H. Wang , Anal. Chem. 2018, 90, 9449. 29969556 10.1021/acs.analchem.8b02096 PMC6372093

[44] C. M. O'Keefe , T. R. Pisanic , H. Zec , M. J. Overman , J. G. Herman , T.‐H. Wang , Sci. Adv. 2018, 4, eaat6459. 30263958 10.1126/sciadv.aat6459 PMC6157960

[45] P. Athamanolap , K. Hsieh , C. M. O'Keefe , Y. Zhang , S. Yang , T.‐H. Wang , Anal. Chem. 2019, 91, 12784. 31525952 10.1021/acs.analchem.9b02344 PMC8645066

[46] E.‐C. Yeh , C.‐C. Fu , L. Hu , R. Thakur , J. Feng , L. P. Lee , Sci. Adv. 2017, 3, e1501645. 28345028 10.1126/sciadv.1501645 PMC5362183

[47] H. Zhu , O. Yaglidere , T.‐W. Su , D. Tseng , A. Ozcan , Lab Chip 2011, 11, 315. 21063582 10.1039/c0lc00358a PMC3073081

[48] T. Gou , J. M. Hu , W. S. Wu , X. Ding , S. F. Zhou , W. B. Fang , Y. Mu , Biosens. Bioelectron. 2018, 120, 144. 30173010 10.1016/j.bios.2018.08.030

