# Supplementary material for: Digital CRISPR/Cas‐Assisted Assay for Rapid and Sensitive Detection of SARS‐CoV‐2
Source: Adv Sci (Weinh). 2021 Jan 12;8(5):2003564. doi: 10.1002/advs.202003564 (PMC7927608; doi:10.1002/advs.202003564)
Supplement: Supplementary file 1 — Supporting Information [file ADVS-8-2003564-s001.pdf]

WILEY-VCH

© 2020 Wiley-VCH GmbH

ADVANCED SCIENCE
Open Access

Supporting Information

for *Adv. Sci.,* DOI: 10.1002/advs.202003564

Digital CRISPR/Cas-Assisted Assay for Rapid and Sensitive Detection of SARS-CoV-2

*Joon Soo Park, Kuangwen Hsieh, Liben Chen, Aniruddha Kaushik, Alexander Y. Trick, and Tza-Huei Wang**

Supporting Information

Digital CRISPR/Cas-Assisted Assay for Rapid and Sensitive Detection of SARS-CoV-2

*Joon Soo Park, Kuangwen Hsieh, Liben Chen, Aniruddha Kaushik, Alexander Y. Trick, and Tza-Huei Wang**

J. S. Park, A. Y. Trick, Prof. T.-H. Wang
Department of Biomedical Engineering, Johns Hopkins University, Baltimore, Maryland 21218, United States
E-mail: thwang@jh.edu

Dr. K. Hsieh, Dr. L. Chen, Dr. A. Kaushik, Prof. T.-H. Wang
Department of Mechanical Engineering, Johns Hopkins University, Baltimore, Maryland 21218, United States

Prof. T.-H. Wang
Institute for NanoBioTechnology, Johns Hopkins University, Baltimore, Maryland 21218, United States

1. Reagents and Materials

Quantitative Synthetic RNA from SARS-Related Coronavirus 2 (NR-52358) was obtained through the BEI Resources Repository, National Institute of Allergy and Infectious Diseases (NIAID), National Institutes of Health (NIH). This preparation includes fragments from the ORF 1ab, Envelope (E) and Nucleocapsid (N) regions. SARS-Related Coronavirus 2, Isolate USA-WA1/2020, heat inactivated (65 °C for 30 min; NR-52350) was deposited by the Centers for Disease Control and Prevention and obtained through the BEI Resources Repository, NIAID, NIH. All Cas12a-guide RNA and DNA oligonucleotides including primers, fluorescently labeled DNA reporter, and fluorescently labeled DNA probes (see Supporting Information Table S1 for sequences) were purchased form Integrated DNA Technologies (IDT; Coralville, IA). Both Cas12a-guide RNA sequences were modified with IDT's proprietary 5' AltR1 and 3' AltR2 modifications. Lyophilized Cas12a-guide RNA was reconstituted in DEPC-treated water (ThermoFisher Scientific, Waltham, MA) at 10 µM. Lyophilized DNA primers, DNA reporter, and DNA probes were reconstituted in nuclease-free water (Promega, Madison, WI) at 100 µM. Reconstituted oligonucleotides were stored at -20 °C. EnGen Lba (Lachnospiraceae bacterium ND2006) Cas12a (Cpf1) (100 µM), Avian Myeloblastosis Virus (AMV) reverse transcriptase (10000 units/mL), and bovine serum albumin (BSA; 20 mg/mL) were purchased from New England BioLabs (Ipswich, MA) and stored at -20 °C. TwistAmp Basic kits were purchased from TwistDx Limited (Maidenhead, United Kingdom) and stored at -20 °C. qScript XLT 1-Step RT-qPCR ToughMix was purchased from Quantabio (Beverly, MA). Tween 20 was purchased from MilliporeSigma (St. Louis, MO) and used without further purification.

2. Experimental Section

2.1. Assembly of Cas12a-assisted RT-RPA mix

Assembly of Cas12a-assisted RT-RPA mix began by resuspending each dried pellet of TwistAmp Basic Reaction mix with 29.5 µL Rehydration buffer to prepare 1.7X rehydrated TwistAmp Basic Reaction mix. A typical Cas12a-assisted RT-RPA mix contained the following: 1X rehydrated TwistAmp Basic Reaction mix, 0.32 µM each of RPA primers,[1] 0.64 µM each of Cas12a-guide RNAs,[1] 4 µM ssDNA reporter, 640 nM EnGen Lba Cas12a, 0.32 U/µL AMV reverse transcriptase, 0.01 mg/mL BSA, 0.1% Tween 20, 14 mM MgOAc, and either synthetic SARS-CoV-2 RNA or heat-inactivated SARS-CoV-2. All components except AMV reverse transcriptase, BSA, Tween 20, MgOAc and synthetic SARS-CoV-2 RNA or heat-inactivated SARS-CoV-2 were first assembled in 1.5 mL protein low-binding microcentrifuge tubes (MilliporeSigma, Burlington, MA), and inside a PCR hood (AirClean Systems, Creedmoor, NC). After a 10 min incubation at room temperature for Cas12a/Cas12a-guide RNAs complex formation, AMV reverse transcriptase, BSA, and Tween 20 were added inside a PCR hood (AirClean Systems, Creedmoor, NC). MgOAc and synthetic SARS-CoV-2 RNA or heat-inactivated SARS-CoV-2 were then added to the mix inside a separate biosafety cabinet (The Baker Company, Sanford, ME) to prevent any carryover contamination.

2.2. Bulk Cas12a-assisted RT-RPA

Bulk Cas12a-assisted RT-RPA was performed in a Bio-Rad CFX96 Touch Real-Time PCR Detection System (Bio-Rad, Hercules, CA). Assembled Cas12a-assisted RT-RPA mix was pipetted into PCR strips (Bio-Rad, Hercules, CA) at a final volume of 10 µL for performing bulk reactions. All bulk reactions were incubated at 42 °C (or the temperatures indicated in Fig. S1) for 60 min, and the fluorescence signals were measured every 1 min. The fluorescence signals measured by the Bio-Rad CFX96 system were displayed without baseline subtraction (i.e., under “No Baseline Subtraction” mode in the CFX Manager Software). A saturated fluorescence intensity was the maximum intensity which the Real-Time PCR Detection System could determine.

2.3. Loading Cas12a-assisted RT-RPA mix into QuantStudio digital chip

To perform digital Cas12a-assisted RT-RPA (deCOViD), assembled Cas12a-assisted RT-RPA mix was first loaded into a commercial QuantStudio 3D Digital PCR 20K Chip v2 (ThermoFisher Scientific, Waltham, MA) by using a QuantStudio 3D Digital PCR Chip Loader and closely following the user guide. Each QuantStudio 3D Digital PCR 20K Chip v2 has a thermally conductive case that securely houses a 10 mm × 10 mm high-density reaction plate containing 20000 reaction wells on its surface; each reaction well is ~700 picoliter in volume for discretizing Cas12a-assisted RT-RPA into independent digital reactions. The loading process began by installing a QuantStudio 3D Digital PCR 20K Chip v2, a QuantStudio 3D Digital PCR Chip Lid v2 (the corresponding adhesive lid with optical window), and a QuantStudio 3D Digital PCR Sample Loading Blade in their respective positions of the loader. Next, 15 µL Cas12a-assisted RT-RPA mix was pipetted in the sample loading port of the loading blade. The loader then automatically moved the loading blade across the chip, simultaneously dispensing the

reaction mix through the loading blade into the reaction wells. Subsequently, several drops of Immersion Fluid were gently (without touching the chip surface) to cover the entire chip. Then, by rotating the loader arm, the chip lid was brought into contact with the chip case and firmly pressed into a tightly sealed assembly. Additional Immersion Fluid was then gently dispensed via a syringe through the fill port in the chip lid while holding the chip and lid assembly at a ~45° angle and allowing air to escape from the fill port, until the chip case contained only an air bubble < 2 – 3 mm in diameter. Finally, the label on the chip lid was firmly pressed over the fill port to establish a fully sealed chip with digitized reaction mix ready for performing digital Cas12a-assisted RT-RPA (deCOViD) – either in end-point format or real-time format.

2.4. End-point digital Cas12a-assisted RT-RPA (deCOViD)

End-point digital Cas12a-assisted RT-RPA (deCOViD) was performed by first heating chips at 42 °C on a ProFlex 2× flat PCR System (Thermo Fisher Scientific, Waltham, MA) and, after heating, measuring the fluorescence signals from the chips via fluorescence microscopy. For heating, each chip was placed within a grid of QuantStudio 3D Digital PCR Chip Adapters that were placed on the sample block of the ProFlex system, which was set to 42 °C. QuantStudio 3D Digital PCR Thermal Pads were laid over the chip adapters before closing the lid of the Proflex system and commencing the reaction. All reactions were performed for either 60 min (e.g., Figure 2) or 30 min (e.g., Figure 3). Of note, we followed the user guide and elevated the chips in the Proflex system during digital Cas12a-assisted RT-RPA (deCOViD), though such an elevation was likely ineffectual to digital Cas12a-assisted RT-RPA (deCOViD). Specifically, the chips were positioned such that their fill ports orient toward the front of the ProFlex system, which was raised to an 11° incline by the underneath QuantStudio 3D Tilt Base. The elevated chips were meant to allow air bubbles formed during thermocycling to float to the elevated fill ports and away from the reaction wells, thus ensuring successful digital PCR. On the other hand, the low, 42 °C reaction temperature of digital Cas12a-assisted RT-RPA (deCOViD) likely minimized air bubbles and thus rendered chip elevation ineffectual.

After heating, each chip was taken from the Proflex system to a fluorescence microscope to measure fluorescence signals from digital Cas12a-assisted RT-RPA (deCOViD). The fluorescence microscope (BX51, Olympus, Japan) is equipped with a collimated LED light source (M625L4-C1; Thorlabs, Inc., Newton, NJ), an Alexa647-compatible filter cube (Semrock Cy5-4040C-OMF; IDEX Health & Science, LLC, Rochester, NY), a 4× magnification objective lens (Olympus UPlanFL N 4×/0.13 NA), and a digital CCD camera (Retiga EXi Fast 1394, QImaging, Canada). The Alexa647-compatible filter cube has a 628 ± 20 nm bandpass excitation filter, a 660 nm dichroic beamsplitter, and a 692 ± 20 nm bandpass emission filter. The CCD camera is connected to a PC and interfaced with QCapture software (QImaging, Canada). For each chip, fluorescence micrographs of 15 distinct regions were taken to cover ~12000 reaction wells. At each region, the shutter was manually opened, a 12-bit fluorescence micrograph was immediately taken under 50 ms exposure, and the shutter was quickly manually closed.

2.5. Real-time digital Cas12a-assisted RT-RPA (deCOViD)

Real-time digital Cas12a-assisted RT-RPA (deCOViD) was performed by heating each chip at 42 °C on a custom heater and concurrently measuring the fluorescence signals from the chips via fluorescence microscopy. The custom heater was made to fit between the fluorescence microscope stage and the 4× magnification objective lens. This was accomplished by assembling the heater with a 50 mm (length) × 50 mm (width) × 3.8 mm (height) standard Peltier module (Custom Thermoelectric, LLC, Bishopville, MD) and a 50 mm (length) × 50 mm (width) × 6 mm (height) heatsink (ATS-CPX050050006-199-C2-R0, Digi-Key, Thief River Falls, MN). The temperature of the heater was controlled by a FTC100D Controller (Accuthermo Technology Corp., Fremont, CA). After the chip was placed on the custom heater and the region to be detected in the chip was selected, the heater was raised to 42 °C and the same region was fluorescently imaged every 1 min for 60 min. At every minute, the shutter was manually opened, a 12-bit fluorescence micrograph was immediately taken under 50 ms exposure, and the shutter was quickly manually closed.

2.6. End-point digital RT-PCR

End-point digital RT-PCR was performed by first assembling an in-house RT-PCR assay using the US CDC-approved SARS-CoV-2 N1 and N2 primers and probes[2]. The 15 μL assay mix contained 1× qScript XLT 1-Step RT-qPCR ToughMix, 500 nM each of CDC N1 primer, 250 nM Cyanine 5 (Cy5)-labeled CDC N1 DNA probe, 750 nM each of CDC N2 primer, 250 nM Fluorescein amidites (FAM)-labeled CDC N2 DNA probe, 1 mg/mL BSA, 0.1% Tween-20, and synthetic SARS-CoV-2 RNA (at varying concentrations). Assembled RT-PCR mix was then loaded into a commercial QuantStudio 3D Digital PCR 20K Chip v2 as described above. The reverse transcription reaction was initiated by chip incubation at 50 °C for 2 min (reverse transcription), followed by PCR amplification at 95 °C for 1 min (hot start), and 50 cycles of 95 °C for 5 s, and 50 °C for 20 s on a ProFlex 2× flat PCR System. After PCR amplification, digital chips were taken from the Proflex system to a fluorescence microscope to measure fluorescence signals from digital RT-PCR as described above. Of note, for consistency with deCOViD, only the fluorescence signals from Cy5-labeled CDC N1 DNA probe was detected by using the same excitation and emission filters as deCOViD.

2.7. Clinical sample testing

Four de-identified clinical nasopharyngeal swabs (including 2 COVID-19 positive samples) in universal transport medium were obtained from the Johns Hopkins Hospital Clinical Microbiology Laboratory in compliance with ethical regulations and the approval of Institutional Review Board (IRB00246027). Viral RNAs from these 4 samples were extracted via ChargeSwitch magnetic beads (Thermo Fisher Scientific, Waltham, MA) following the manufacturer's instructions. Ten μL of each sample was used as the input for extraction and eluted with 20 μL elution buffer. Prior to testing with the bulk CRISPR/Cas-assisted assay and deCOViD, eluted RNAs from these samples were screened for SARS-CoV-2 by the in-house RT-PCR assay (with qScript XLT 1-Step RT-qPCR ToughMix and US CDC-approved SARS-CoV-2 primers and probes) performed in a Bio-Rad CFX96 Touch Real-Time PCR Detection System under real-time detection mode. RT-qPCR is performed at 50 °C for 2 min (reverse

transcription), followed by 95 °C for 1 min (hot start) and 50 cycles of 95 °C for 5 s and 50 °C for 20 s, with the fluorescence measured at 50 °C of each cycle.

2.8. Data processing and statistical analysis

Data acquisition from fluorescence micrographs and subsequent data analysis were performed using ImageJ (1.52p), MATLAB (R2019b), Microsoft Excel 365, and Origin 2018. Region of interest (ROI) from each fluorescence micrograph was defined with ImageJ. Fluorescence intensities from the reaction wells in the QuantStudio 3D Digital PCR Chip were measured with ImageJ and MATLAB. Downstream data analyses including statistical analysis, plotting, and data fitting were performed with Excel and Origin.

For end-point digital analysis, each fluorescence micrograph was first converted to 8-bit grayscale in ImageJ. Using the publicly available "AdaptiveThreshold" plugin function in ImageJ, the "Mask" image was created. Subsequently, the ROIs from each "Mask" image were defined using the "Analyze particles" function in ImageJ and saved for downstream fluorescence intensity measurements. Each ROI file from ImageJ was then converted into a MATLAB structure using the publicly available MATLAB program, "ReadImageJROI". A custom-built MATLAB program was developed for fluorescence intensity measurements using fluorescence micrographs, the "Mask" images, and corresponding ROIs. The program measured fluorescence intensities of each reaction well from each of 15 micrographs and counted the number of reaction wells with the fluorescence intensity below and above threshold for quantifying negative and positive wells, respectively. The threshold value was empirically set at 15000 and sufficiently higher than non-specific signals from no-template controls, which could sometimes reach ~10000. The program then combined 15 measurements for calculating the percent positive (*i.e.*, the number of positive reaction wells divided by the total number of reaction wells) of each digital reaction and extracting the end-point fluorescence intensities of positive wells.

For real-time digital analysis, a single ROI was defined from a micrograph at 1 min as described above. With a single ROI, fluorescence intensities of reaction wells in micrographs at each min were measured using "Multi Measure" function in ROI manager. The measurements were exported, and real-time curves from individual reaction wells were plotted via Origin. The time-to-positive values were then calculated via custom-built MATLAB program for each positive well, a time point where fluorescence intensity first exceeds the threshold.

All downstream data analyses were performed via Excel and Origin. The time-to-positive (i.e., Figure 2e) and the signal-to-background ratio (i.e., Figure 2f) from each RNA concentration were calculated from triplicated results of bulk experiment (i.e., $n = 3$) and collected positive signals ($n > 3$) from a single digital chip, and presented as mean ± SD. For the signal-to-background ratio calculation, the end-point fluorescence intensity at 30 min (F) was divided by the initial fluorescence intensity at 1 min ($F_{initial}$) for both bulk and digital Cas12a-assisted RT-RPA reaction. The calculated $F/F_{initial}$ values from each synthetic SARS-CoV-2 RNA concentration were plotted as a scatter plot. For sensitivity comparison between bulk and digital

Cas12a-assisted RT-RPA reaction with both synthetic SARS-CoV-2 RNA (i.e., Figure 3a) or heat-inactivated SARS-CoV-2 (i.e., Figure 3b), the end-point fluorescence intensities at 30 min in bulk reaction and the percent positives in digital reaction were plotted, and linearly fitted via Origin. All RNA concentrations were tested in triplicate (i.e., n = 3) with both bulk reaction and digital reaction. The data are presened as mean ± SD.

3. Supporting Tables and Figures

Table S1. Sequences of RPA primers, Cas12a-guide RNAs, and ssDNA reporter.

**Description**	**Nucleic Acid Sequence (5’ → 3’)**
RPA Forward primer[1]	AGGCAGCAGTAGGGGAACTTCTCCTGCTAGAAT
RPA Reverse primer[1]	TTGGCCTTTACCAGACATTTTGCTCTCAAGCTG
Lba Cas12a-guide RNA1[1]	/AltR1/UAAUUUCUACUAAGUGUAGAU**CAUCACCGCCAUUGCCAGCC**/AltR2/
Lba Cas12a-guide RNA2[1]	/AltR1/UAAUUUCUACUAAGUGUAGAU**UUGCUGCUGCUUGACAGAUU**/AltR2/
ssDNA reporter	/Alex647N/TTATT/IAbRQSp/
CDC N1 Forward primer	GACCCCAAAATCAGCGAAAT
CDC N1 Reverse primer	TCTGGTTACTGCCAGTTGAATCTG
CDC N1 probe	/Cy5/ACCCCGCATTACGTTTGGTGGACC/IAbRQSp/
CDC N2 Forward primer	TTACAAACATTGGCCGCAAA
CDC N2 Reverse primer	GCGCGACATTCCGAAGAA
CDC N2 probe	/FAM/ACAATTTGCCCCCAGCGCTTCAG/IAbFQSp/

Note. Bolded: Target regions in crRNAs, Abbreviations: LbCas12a, New England Biolabs’ EnGen Lba Cas12a from *Lachnospiraceae bacterium* ND2006; AltR1 and AltR2, Integrated DNA Technologies’ proprietary Alt-R modifications; IAbRQSp, Iowa Black RQ; IAbFQSp, Iowa Black FQ.

Table S2. Assay format, time, and limit of detection for RNA of CRISPR/Cas-assisted SARS-CoV-2 detection assays.

Assay	Amplification	Cas Effector	Detection	Number of Steps	Assay Time (min)	Limit of Detection (cps/μL)[a]	RNA Target[b]	Status & Citation in Main Text
deCOViD	RT-RPA	LbCas12a	Digital Fluorescence	1	15 – 30	1	Synthetic RNA (NIAID BEI Resources)	This Work
DETECTR	RT-LAMP	LbCas12a	Fluorescence	2	30 – 40	10	IVT RNA	Published [22]
			Lateral Flow	3	35 – 45	10		
AIOD-CRISPR	RT-RPA	LbCas12a	Fluorescence	1	40	0.18	IVT RNA	Published [23]
CASdetec	RT-RAA	AaCas12b	Fluorescence	1[c]	40 – 60	5	IVT RNA	Published [24]
CRISPR-FDS	RT-PCR	LbCas12a	Fluorescence	2	> ~57[d]	0.1	Extracted RNA	Published [25]
	RT-RPA			2	40	0.1		
C. Lucia, et al.	RT-RPA	LbCas12a	Fluorescence	2	< 120	10	IVT RNA	BioRxiv Prepeint [26]
VaNGuard	RT-LAMP	Various Cas12a	Fluorescence	2	25	8	IVT RNA	BioRxiv Prepeint [27]
			Lateral Flow	3	25 – 30	8	IVT RNA	
CRISPR/Cas12a-NER	RT-RAA	FnCas12a[e]	Fluorescence	2	45	10	IVT RNA	Published [28]
CONAN	RT-LAMP	EcCas3	Fluorescence	2	30	< 100 cps[f]	Extracted RNA	MedRxiv

			Lateral Flow	3	32	< 100 cps[f]		Prepeint [29]
STOP	RT-LAMP	AaCas12b	Fluorescence	1	40	2	Genome Standards	Published [31]
			Lateral Flow	2	70	2		
SHERLOCK	RT-RPA	LwCas13a	Fluorescence	2	55 – 85	3	Extracted RNA	Published [32]
			Lateral Flow	2	55	3		

Note. [a] Limit of detection was calculated by authors of this work when necessary. [b] Assays that measure the limit of detection of DNA plasmids are excluded. [c] A brief centrifugation step is omitted from the number of steps. [d] Assay time was calculated by authors of this work by summing RT-PCR time without ramp time (55 °C for 10 min, 98 °C for 2 min, 35 cycles at 98 °C for 10 s, 60 °C for 10 s, and 72 °C for 15 s, 72 °C for 5 min). [e] The authors provide the following reference for the Cas12a used in their work: Creutzburg S.C.A., Wu W.Y., Mohanraju P. Good guide, bad guide: spacer sequence-dependent cleavage efficiency of cas12a. Nucleic Acids Res. 2020; 48:3228 – 3243. [f] The reaction volume cannot be determined by the authors of this work. Abbreviations: RT-RPA, reverse transcription recombinase polymerase amplification; RT-LAMP, reverse transcription loop mediated isothermal amplification; RT-RAA, reverse transcription recombinase-aided amplification; IVT, in vitro transcription; LbCas12a, Cas12a from *Lachnospiraceae bacterium* ND2006; AaCas12b, Cas12b from *Alicyclobacillus acidiphilus*; FnCas12a, Cas12a from *Francisella tularensis* subsp. Novicida; EcCas3, Cas3 from *Escherichia coli*; LwCas13a, Cas13a from *Leptotrichia wadei*; NIAID, National Institute of Allergy and Infectious Diseases

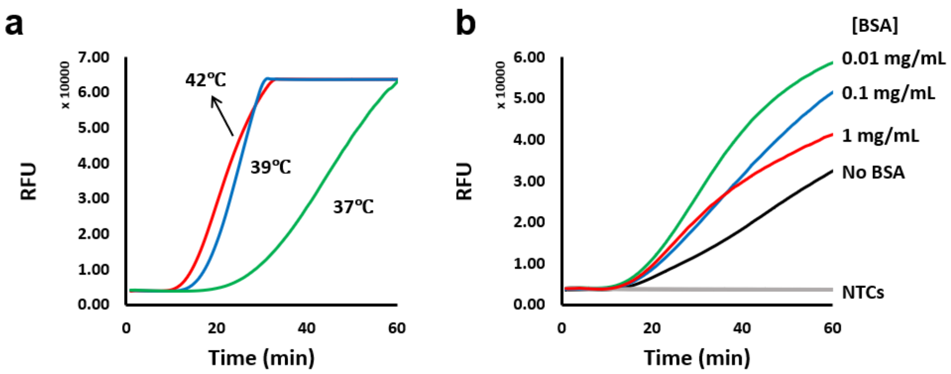

Figure S1. Optimization of one-pot CRISPR/Cas12a-assisted RT-RPA assay. (a) Optimization of reaction temperature with 200 genome equivalent (GE)/μL RNA; 37 ℃ - an optimal temperature for CRISPR/Cas12a reaction, 39 ℃ - an optimal temperature for RPA reaction, 42 ℃ - a minimum optimal temperature for reverse transcriptase activity. Reaction temperature of 42℃ shows the earliest signal increase, suggesting that the reverse transcription is the rate-limiting step in our assay. (b) BSA as an additive; With 100 GE/μL RNA, addition of BSA improves assay performance compared to the reaction without BSA. Increasing BSA concentration shows decreasing trend of the assay performance. 0.01 mg/mL BSA was selected as a final reaction condition.

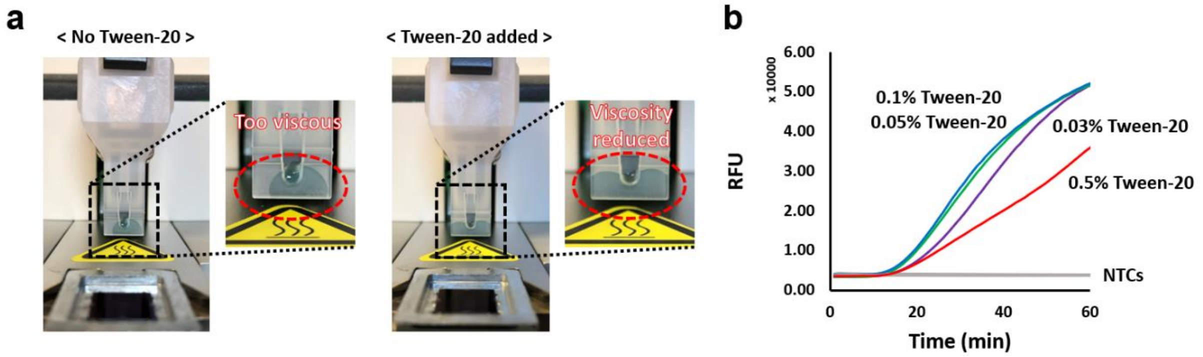

Figure S2. Tween-20 as an additive for deCOViD. Due to the viscosity of the one-pot CRISPR/Cas12a-assisted RT-RPA reaction mix, addition of Tween-20 is necessary for allowing the assay to be compatible with the QuantStudio 3D Digital PCR 20K Chip. (a) Without Tween-20, highly viscous reaction mix is trapped in the middle of the Sample Loading Blade, requiring manual tap-down to bring down the sample. Addition of Tween-20 shows an evenly dispensed reaction mix in the Sample Loading Blade. (b) 0.1% Tween-20 is shown to be the maximum concentration while retaining the assay performance.

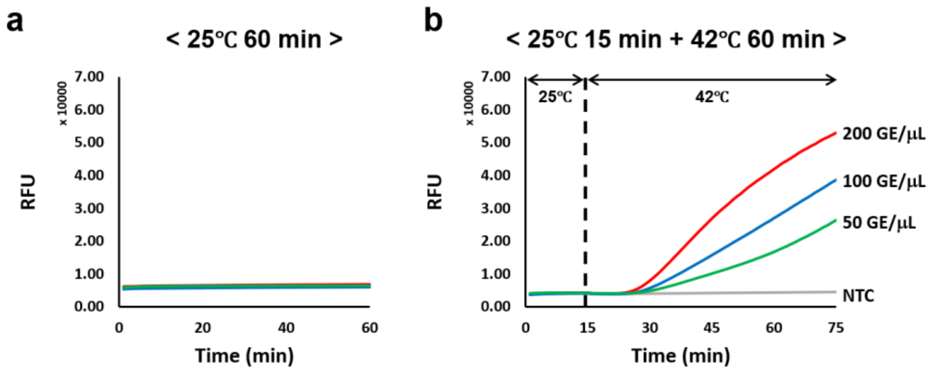

Figure S3. Inactivity of one-pot CRISPR/Cas12a-assisted RT-RPA assay at room temperature. (a) The assay performed at room temperature (25 ℃) for 60 min shows indistinguishable fluorescence signals between samples containing synthetic SARS-CoV-2 RNA and the no template control (NTC). (b) Pre-incubation of the one-pot CRISPR/Cas12a-assisted RT-RPA assay at room temperature (25 ℃) for 15 min exhibits a comparable signal increase pattern without pre-incubation, suggesting a minimal influence of ~5 min hands-on time for assay digitization into the QuantStudio digital chip on the quantitative accuracy of deCOViD.

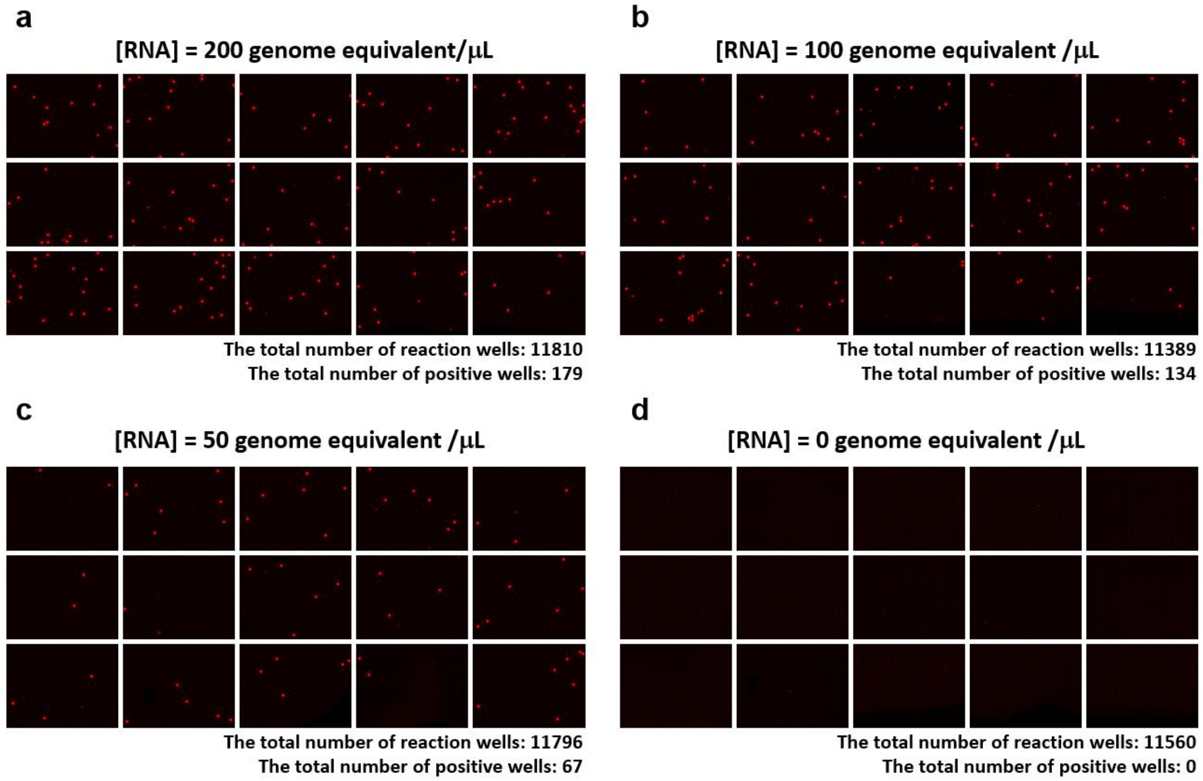

Figure S4. End-point fluorescence micrographs of QuantStudio digital chip. Fluorescence micrographs of 15 distinct regions with (a) 200 genome equivalent (GE)/μL, (b) 100 GE/μL, (c) 50 GE/μL, and (d) 0 GE/μL RNA show that the number of positive wells in each chip reduces with decreasing target concentration.

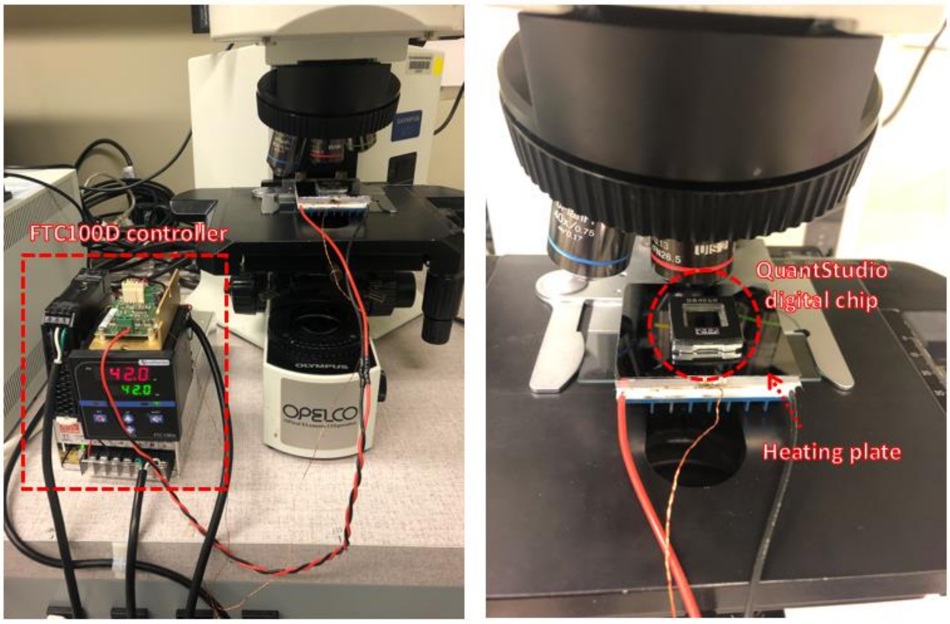

Figure S5. Custom-built miniature heater for real-time deCOViD. We employ custom-built heater to incubate the QuantStudio digital chip while placing it on the microscope. Fluorescence images of one region of digital chip (consisting of ~800 wells) are taken every 1 min for real-time detection.

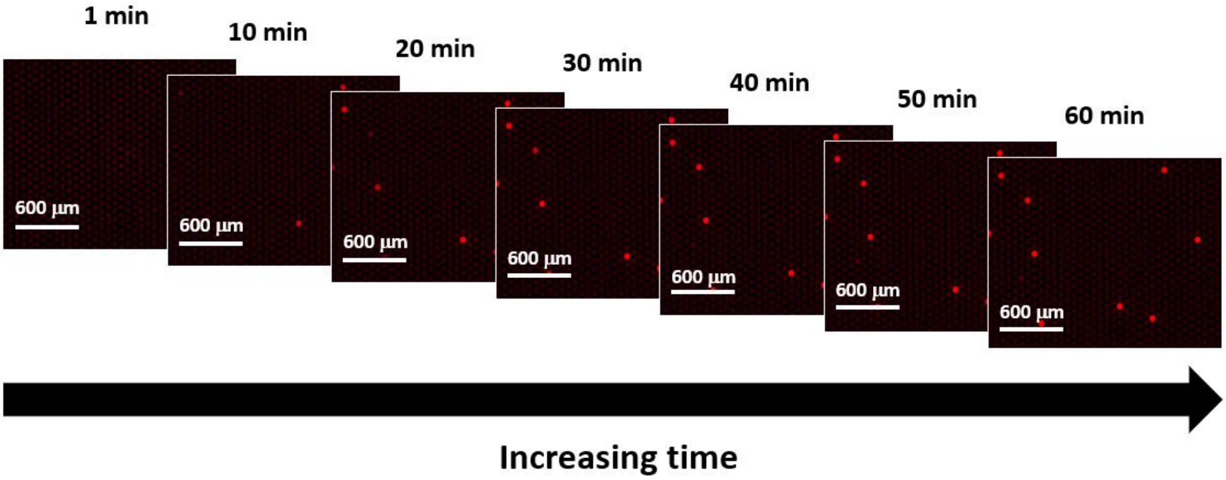

Figure S6. Real-time fluorescence micrographs at increasing time points. For real-time analysis of deCOViD, fluorescence micrographs of a single region (~800 digital wells) are taken every 1 min.

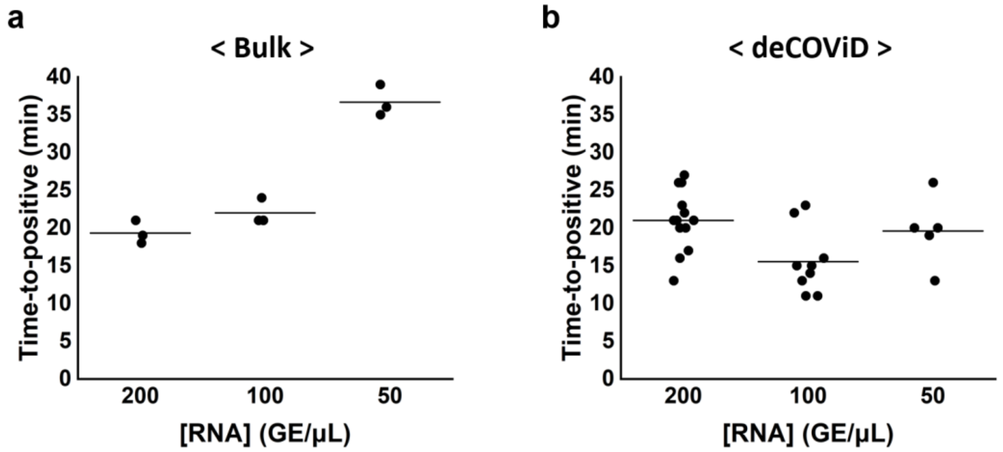

Figure S7. Time-to-positives for bulk assay and deCOViD. (a) Triplicated time-to-positive results from bulk assay with decreasing target concentration show a sharp increase of time-to-positive below 100 genome equivalent (GE)/µL. (b) Time-to-positives of deCOViD analyzed from ~800 digital wells in each QuantStudio digital chip with varying target concentrations. While the number of positive wells reduces with decreasing target concentration, single-molecule detection of digital assay allows average time-to-positives to be consistent regardless of target concentration. Moreover, the heterogeneity of time-to-positives in each target concentration indicates that < 15 min is sufficient for qualitative detection of SARS-CoV-2 RNA target.

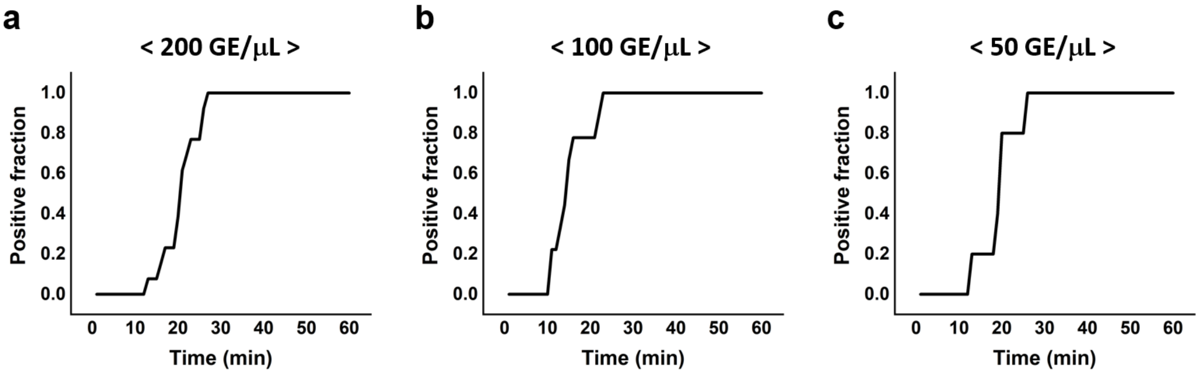

Figure S8. Positive fractions in deCOViD across three RNA concentrations. Positive fractions with (a) 200 genome equivalent (GE)/μL, (b) 100 GE/μL, and (c) 50 GE/μL RNA show that every positive well can be detected within 30 min.

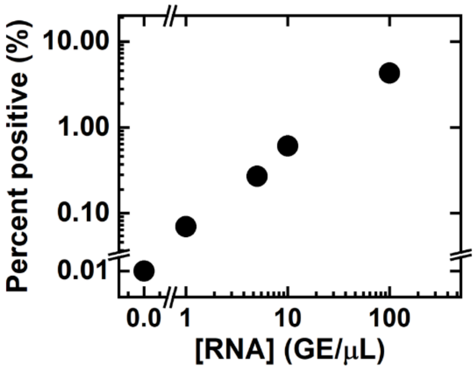

Figure S9. Detection of synthetic SARS-CoV-2 RNA via digital RT-PCR. Digital RT-PCR is achieved by digitizating an in-house RT-PCR assay containing US CDC-approved primers and probes into QuantStudio digital chips. The results show a linear relationship between the RNA concentration (in genome equivalent (GE)/µL) and the percent positive (*i.e.*, the fraction of positive reaction wells of the total number of reaction wells). Digital RT-PCR shows ~4 – 5-fold higher percent positive than deCOViD, which may be due to different copy numbers of N gene fragments with two different sequences targeted by the RT-PCR assay and deCOViD in synthetic SARS-CoV-2 RNA (NIAID BEI Resources).

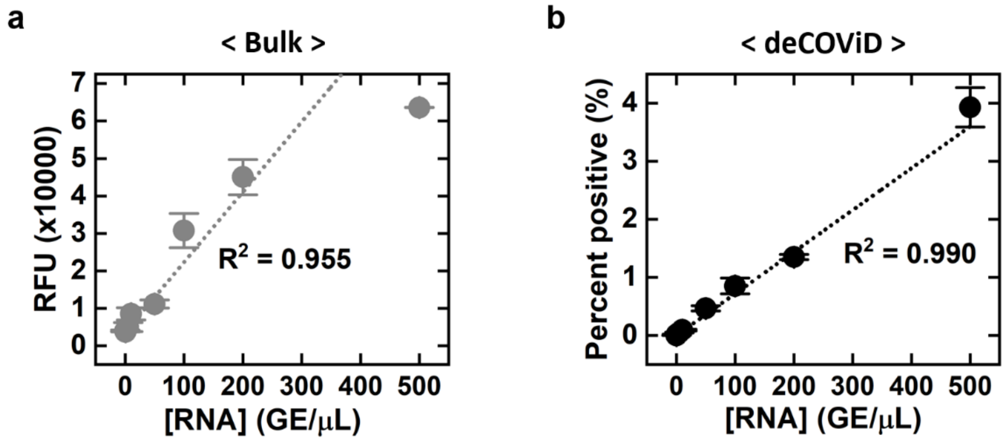

Figure S10. Detection of synthetic SARS-CoV-2 RNA via bulk assay and deCOViD at 30 min. (a) Bulk assay shows saturated end-point fluorescence intensity in between 200 and 500 genome equivalent (GE)/µL, indicating the limit of quantification at this range. (b) As deCOViD relies on the number of positive wells rather than the end-point signal for quantification, linear relationship between target concentration and percent positive continues beyond 500 GE/µL, emphasizing the wider dynamic range of quantitative detection in deCOViD compared to the bulk assay. Date presented as mean ± SD, n = 3.

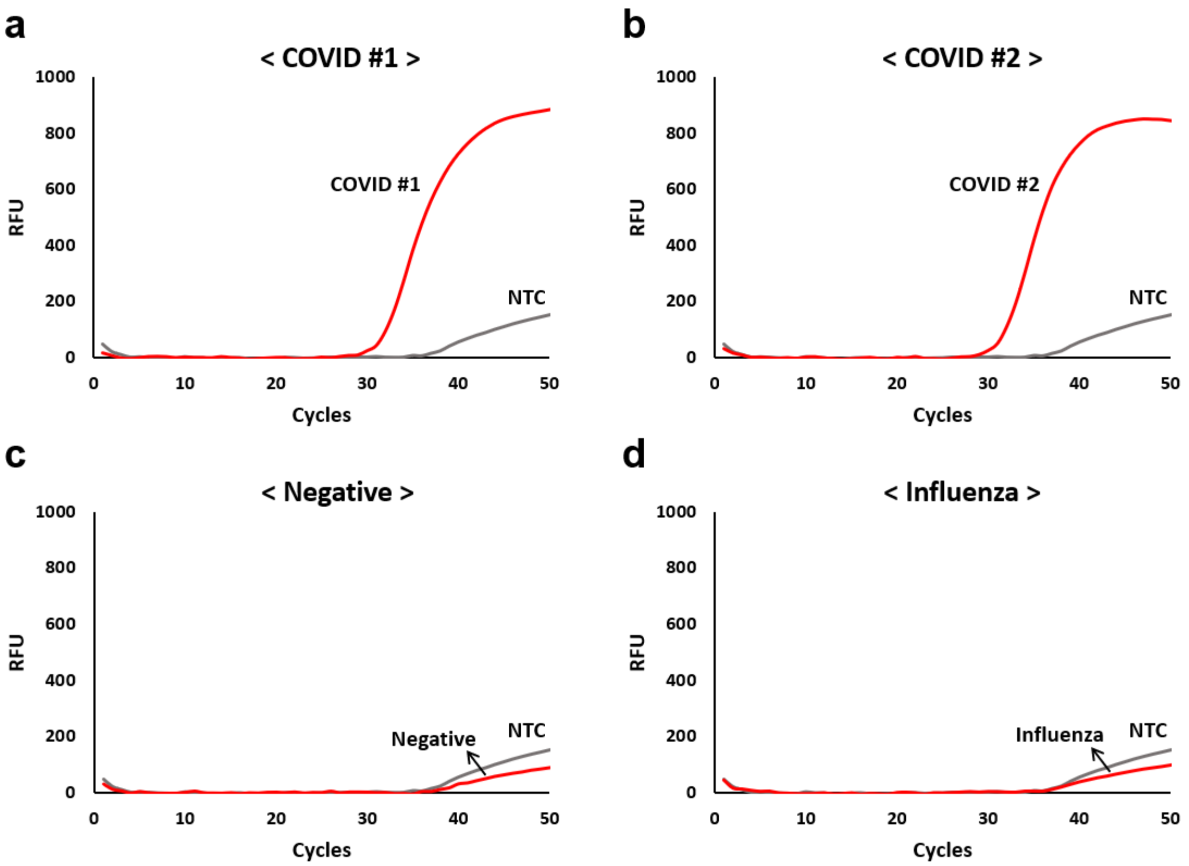

Figure S11. Detection of clinical nasopharyngeal swab samples via in-house RT-qPCR assay. Four RNA extracts from de-identified clinical nasopharyndeal swab samples were screened via an in-house RT-qPCR assay using US CDC-approved primers and probes. Two SARS-CoV-2 positive samples, (a) COVID #1 and (b) COVID #2, yield robust amplification curves, whereas (c) a SARS-CoV-2 negative and (d) an Influenza sample show amplification curves that are identical to the no-template control (NTC).

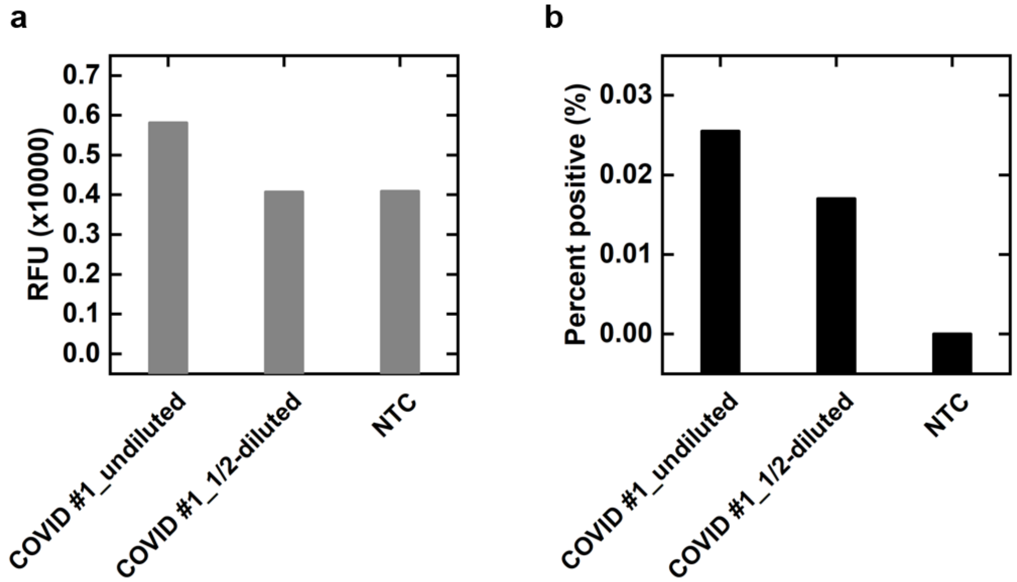

Figure S12. Sensitivity comparison between bulk CRISPR/Cas-assisted RT-RPA assay and deCOViD using a diluted clinical nasopharyngeal swab sample. (a) The bulk assay can only differentiate the undiluted SARS-CoV-2 positive sample from the no template control (NTC). (b) In contrast, deCOViD, can detect both the undiluted and the 2-fold-diluted SARS-CoV-2 positive samples.

4. References

[1] X. Ding, K. Yin, Z. Li, R. V. Lalla, E. Ballesteros, M. M. Sfeir, C. Liu, *Nat. Commun.* **2020**, *11*, 4711.

[2] X. Lu, L. Wang, S. K. Sakthivel, B. Whitaker, J. Murray, S. Kamili, B. Lynch, L. Malapati, S. A. Burke, J. Harcourt, A. Tamin, N. J. Thornburg, J. M. Villanueva, S. Lindstrom, *Emerging Infect. Dis.* **2020**, *26*, 1654.
